# CONNECTING AGING RURAL VETERANS WITH TELE-GERIATRIC MENTAL HEALTH: REFERRING PROVIDERS’ SURVEYED PERSPECTIVE

**DOI:** 10.1093/geroni/igad104.1456

**Published:** 2023-12-21

**Authors:** Chalise Carlson, Marika Humber, Ranak Trivedi, Amanda Peeples, Althea Lloyd, Christine Gould

**Affiliations:** VA Palo Alto Health Care System, Palo Alto, California, United States; VA Palo Alto Health Care System, Palo Alto, California, United States; Stanford University, Stanford, California, United States; Corporal Michael J. Crescenz VA Medical Center, Philadelphia, Pennsylvania, United States; VISN 7, Clinical Resource Hub, VA Atlanta Health Care System, Decatur, Georgia, United States; VA Palo Alto Health Care System, Palo Alto, California, United States

## Abstract

Providing comprehensive, individualized care to older Veterans with complex needs is particularly challenging in rural areas. Geriatric mental health specialists are scarce but needed for care including psychiatric medication and behavior management, consultation on complex comorbidities, cognition, and caregiver support and education. Tele-geriatric mental health services were initially developed in one VA regional telehealth hub and then expanded to three more regional telehealth hubs. Referring providers (N = 37) to tele-geriatric mental health services during a 1-year period were surveyed via REDCap. Providers came from varied settings in the 4 VA regions with 76% serving rural Veterans. Referring providers included prescribers (N = 26; pharmacists, physicians, physician assistants, nurse practitioners and advanced practice nurses) and non-prescribers (N = 11; psychologists, social workers). Most worked in Patient Aligned Care Teams, mental health clinics, or Community Living Centers and reported geriatric training and comfort with geriatric patients. When asked about their perception of the impact of the services, most providers believed that patients (91.9%) and care teams (94.6%) benefitted from the telehealth services and would recommend the services (94.6%). Most followed through on recommendations (83.7%) and believed the service increased access to geriatric mental health (89.2%). Open-ended feedback included gratefulness for providing care to aging rural Veterans lacking resources. These services are appreciated by providers and may facilitate access to specialized mental health care particularly in rural areas. Having access to specialists to assist with the care of patients with complex needs may ease the care coordination needed by referring providers.

